# Infection of a Free-Living Wild Boar (*Sus scrofa*) with a Bacterium from the *Mycobacterium kansasii* Complex

**DOI:** 10.3390/ani12080964

**Published:** 2022-04-08

**Authors:** Łukasz Radulski, Monika Krajewska-Wędzina, Marek Lipiec, Krzysztof Szulowski

**Affiliations:** National Veterinary Research Institute, 24-100 Puławy, Poland; monika.krajewska@piwet.pulawy.pl (M.K.-W.); mlipiec@piwet.pulawy.pl (M.L.); kszjanow@piwet.pulawy.pl (K.S.)

**Keywords:** mycobacteriosis, wild boar, *Mycobacterium kansasii* complex

## Abstract

**Simple Summary:**

Mycobacteriosis is a collective term for diseases caused by nontuberculous mycobacteria. Wild animals are a frequent source of mycobacteria infection in farm animals and humans; therefore, it is important to monitor the presence of these pathogens in free-living mammals. We isolated bacterium belonging to *Mycobacterium kansasii* complex from a submandibular lymph node obtained from a wild boar. This mycobacterium is a common cause of severe human lung diseases and is rarely responsible for animal diseases; therefore, its presence in the wild animal population is of great concern. The animal was apparently healthy, and we did not find any internal organ lesions despite the abundant growth of tissue-isolated bacteria on media. Thanks to our research, the specificity of wild boar mycobacteriosis caused by MKC will be better known.

**Abstract:**

The most numerous group of bacteria in the genus *Mycobacterium* is the nontuberculous mycobacteria. Currently, over 200 species of bacteria have been classified as belonging to this group, of which approximately 30 are pathogenic to humans and animals. *Mycobacterium kansasii* complex numbers among these pathogenic species. The submandibular lymph nodes of a wild boar shot by a hunter were examined in order to confirm or exclude infection with bacteria of the genus *Mycobacterium*. In culture, a bacterial isolate was obtained after 12 days of incubation on Petragnani and Stonebrink media. A multiplex PCR clearly indicated that the isolate was a nontuberculous mycobacterium. The results of species identification attempts via both molecular biology methods and mass spectrometry confirmed that the isolated strain belonged to MKC. The described case of a wild boar infection with MKC is the first documented case in Poland and only the second in Europe, and in confirming the presence of this pathogen among free-living animals, this report implies that MKC is of great concern. Our research elucidates some specifics of wild boar mycobacteriosis and may be used to instill awareness in the public of the dangers of dressing hunt prey or consuming its meat in ignorance of safe procedures, which can contribute to the transmission of the pathogen to humans.

## 1. Introduction

According to the currently valid classification, acid-fast mycobacteria belong to the Schizomycetes class, Actinomycetales order, Mycobacteriaceae family, and *Mycobacterium* genus [1]. These bacteria are some of the most dangerous pathogens threatening both animal and human health and life [2]. Mycobacteria can be divided into typical species, causing tuberculosis and belonging to the *Mycobacterium tuberculosis* complex (MTBC) group, and nontuberculous mycobacteria (NTM) [3]. The single exception to this duplex is *M. lapreae*, the causative agent of leprosy, which is classified separately [4]. The most numerous group of mycobacteria is NTM. Currently, over 200 bacteria have been classified as belonging to this group, of which approximately 30 are pathogenic to humans and animals [5]. However, these numbers are frequently updated because new species are recorded each year. Along with the increase in the number of described species in the world, there is a global upward trend in the reported cases of mycobacterial infections, both in humans and animals [6]. Free-living animals such as wild boar are frequently the host of NTM and MTBC [7]. Their free migration renders them effective in spreading the disease associated with bacteria from the genus *Mycobacterium*. In the regions of southern Spain with highest cattle bTB prevalence, wildlife species such as wild boar (*Sus scrofa*) and red deer (*Cervus elaphus*) show high prevalence of bTB, which suggests that the disease is shared between domestic and wild hosts [8].

The diseases caused by NTM are called mycobacteriosis. They may include lungs, lymph nodes, skin, and subcutaneous tissue diseases as well as peritonitis. One of the most dangerous species of nontuberculous mycobacteria is *M. kansasii*, because mycobacteriosis caused by this mycobacterium can lead to the death of the animal, and untreated in humans, it is fatal in over 50% of those infected [9].

*M. kansasii* was described for the first time in 1953. This species has been isolated and identified as a pathogen of free-living animals and humans [10]. It is often found in water and soil, which may be its natural habitat [11]. Like other members of the genus *Mycobacterium*, *M. kansasii* is a Gram-positive organism, immobile and non-spore-forming [12]. In laboratory conditions, it grows at a temperature of 32 °C to 42 °C. Colonies can range in shape and color. *M. kansasii* is a photochromogenic microorganism, belonging to group I according to Runyon’s classification. When exposed to visible light, they usually turn orange because of the production of carotenoids, mainly β-carotene (approximately 85% of carotenoids produced) and lycopene (15%) [11]. Among the species *Mycobacterium kansasii*, seven subtypes have been previously reported based on the PCR and the restriction fragment length polymorphism of the gene hsp65. Only very recently, it has been proposed that the hitherto existing *Mycobacterium kansasii* subtypes (I–VI) should be elevated, each to a species rank, and form the *Mycobacterium kansasii* complex (MKC). Consequently, the former *M. kansasii* subtypes have been denominated as *Mycobacterium kansasii* (former type I), *Mycobacterium persicum* (II), *Mycobacterium pseudokansasii* (III), *Mycobacterium innocens* (V), and *Mycobacterium attenuatum* (VI) [6].

Animal diseases are rarely caused by *M. kansasii*, which is much more often isolated from humans. The estimated incidence of *M. kansasii* in humans ranges from 2 to 118 cases per 1,000,000 people, rising to 5320 in the population of people with AIDS [13]. Human-to-human transmission is thought not to occur [14]. Although there is wide geographic variation in the incidence of infections with this pathogen, in many regions, it ranks first as the cause of non-tuberculous mycobacterial lung disease in the HIV-seronegative population and second as the cause of generalized infection among HIV-seropositive patients [12]. Along with *M. avium* complex mycobacteria, this bacterium is also one of the most frequently isolated NTM species from clinical specimens around the world, and *M. kansasii* ranks among the most clinically relevant isolated species [14]. Clinical syndromes of pneumonia and radiological findings in the lungs due to *M. kansasii* infections are mostly indistinguishable from those of MTBC, definitive diagnosis thus requiring microbiological confirmation [14]. The final diagnosis of the etiological factor is of key importance in deciding on the appropriate anti-bacillus therapy. While mycobacteriosis caused by *M. kansasii* is most often located in the lungs [15,16,17], there are also reports of extrapulmonary diseases caused by this microbe [18,19,20]. Extrapulmonary disease caused by *M. kansasii* most often affects the skin, lymph nodes, and genitourinary and musculoskeletal systems.

The aim of the study was to confirm or rule out the infection of free-living animal with bacteria belonging to the *Mycobacterium* genus and to determine the species of the isolated strains. The results of this paper may contribute to making the affected sections of society aware of the dangers of dressing hunt prey or consuming its meat in ignorance of safe procedures.

## 2. Materials and Methods

### 2.1. Collection of Samples

The submandibular lymph nodes of a male wild boar from the southern part of Poland were examined. The animal was shot by hunters and showed no signs of disease. Samples were sent to the National Veterinary Research Institute in Poland (NVRI) to evaluate the prevalence of tuberculosis and mycobacteriosis in free-living animal populations. The tested sample was 1 out of 35 obtained in 2019 from free-living wild boars.

### 2.2. Lesion Assessment and Mycobacterial Isolation

The first stage of the research was an anatomopathological examination to locate and assess the nature of lymph nodes lesions in the structure of the submandibular lymph nodes. Subsequently, 15 mL of oxalic acid was added to approximately 3 g of finely cut tissue which had been homogenized for 3 min in MiniMix tissue homogenizer (Interscience, Saint-Nom-la-Bretèche, France) and placed in a sterile filter bag. The filtrate was poured into a Falcon tube and incubated at 37 °C for 20 min to improve the efficiency of the test sample decontamination procedure. After incubation, the sample was centrifuged at 3570 RCF for 10 min, then washed with sterile physiological saline and centrifuged again at 3570 RCF for 10 min. The rinsing step was performed twice. The prepared material was plated on four Stonebrink and four Petragnani media manufactured in NVRI, and then incubated at 37 °C ± 2 °C.

### 2.3. Strain Generic Identification

**Figure 1:**
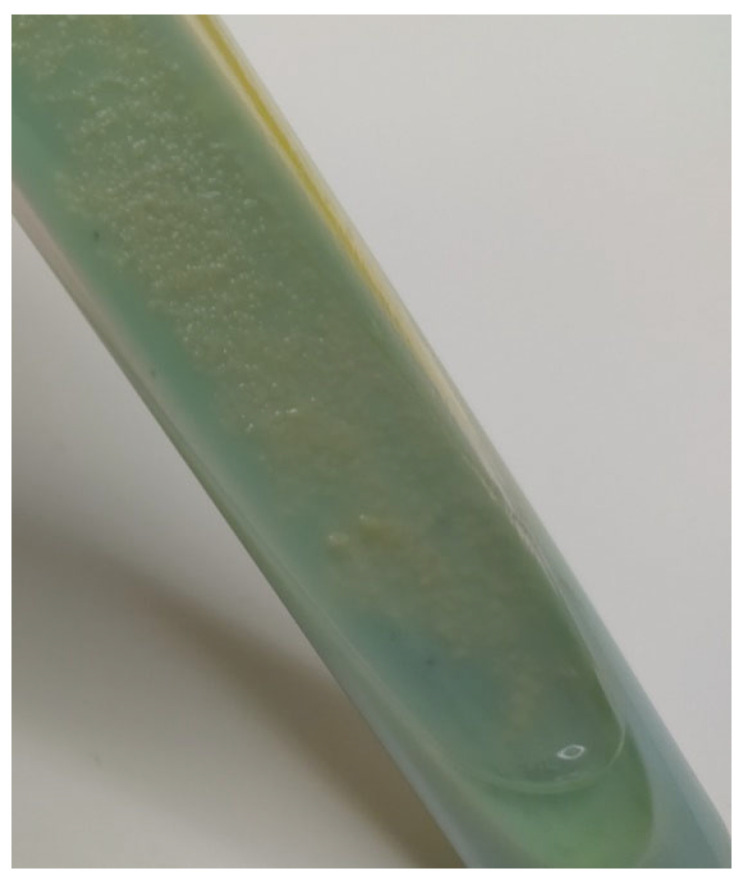
MKC growth on Petragnani medium.

In order to control the acid resistance of the strain, Ziehl–Neelsen staining was carried out (Figure 1) [21]. To determine whether a given strain belonged to the group of tuberculous or the group of nontuberculous mycobacteria, a multiplex PCR was performed (Table 1, Table 2 and Table 3). In this method, two pairs of primers were used, which allowed for the simultaneous amplification of two different DNA fragments. The primers were constructed by European Union Reference Laboratory for bovine tuberculosis in Madrid and synthesized by Eurofins Genomics company (Eurofins Genomics, Ebersberg, Germany). The first DNA fragment comprised a 1030 bp sequence of a gene encoding the 16S rRNA characteristic of each bacterium belonging to the *Mycobacterium* genus. The second sequence was a 372 bp DNA fragment of the MPB70 protein gene, the presence of which is characteristic only of tuberculous bacilli. The final stage of the identification method was 2% agarose gel electrophoresis (70 V, 80 min). The appearance of the reaction product with a length of 1030 bp indicated that the tested strain was a member of the genus *Mycobacterium*. The presence of the second reaction product with a length of 372 bp indicated that the analyzed strain affiliated to the MTBC. The results of the multiplex PCR test informed the selection of appropriate species identification methods, because these methods differ depending on the type of mycobacterium being tested for.

### 2.4. Strain Species Identification

The GenoType Mycobacterium CM test (Hain Lifescience, Nehren, Germany) was used for species identification, following the manufacturer’s instructions. The Hain Lifescience CM is a qualitative test designed for in vitro diagnosis of nontuberculous mycobacteria. It is based on a PCR technique that targets the unique sequences of the 23S rRNA gene. The test uses strips coated with specific probes complementary to the amplified nucleic acids. After chemical denaturation, single-stranded amplicons bind to the probes and hybridization occurs. On each strip, there are 14 areas of specific DNA sections characteristic of each NTM species and three controls. The color of the strip indicates the presence or absence of a DNA sequence contained in the genome of a particular bacterium in the tested sample [22]. For the proper conduct of the test, 5 µL of 20–30 ng/µL high-quality DNA with the lowest possible fragmentation is required. Therefore, the Genomic Mini AX Bacteria kit (A&A Biotechnology, Gdańsk, Poland) with gravity-operated DNA purification columns was used for the extraction of nucleic acids, which yielded high-quality nucleic acids. In order to validate the results, two diagnostic methods were used. The method used to confirm the results of the Hain Lifescience CM test was MALDI-TOF mass spectrometry with a Bruker Biotyper System (Bruker, Billerica, MA, USA). For this purpose, an amount of bacterial biomass corresponding to 2 full volumes of a calibrated 1 µL inoculation loop was added to a test tube containing 50 µL of trifluoroacetic acid (TFA). The prepared sample was incubated for 30 min at room temperature, and then it was diluted tenfold by adding 450 µL of sterile distilled water. Afterwards, 1 µL of the diluted mixture was applied to a MALDI plate. After the applied sample was dry, 1 µL of matrix was added and analyzed [23].

## 3. Results

**Figure 2:**
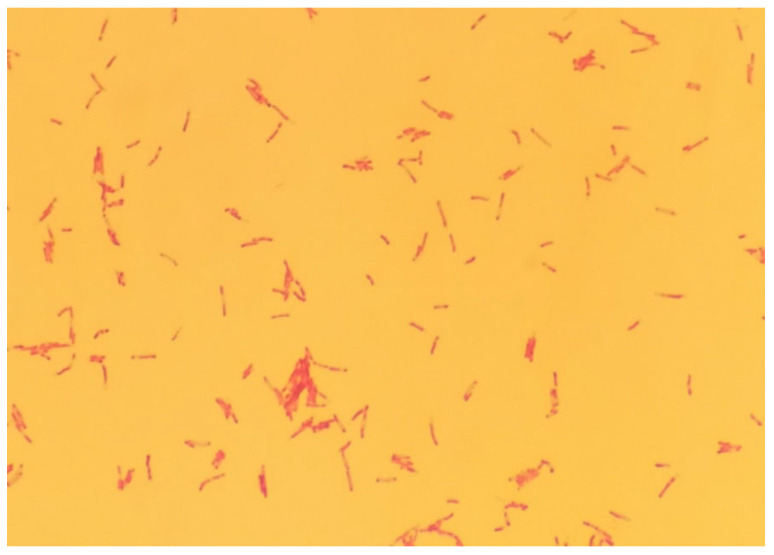
Acid-fast rods (Ziehl-Neelsen staining, 1000×).

**Figure 3:**
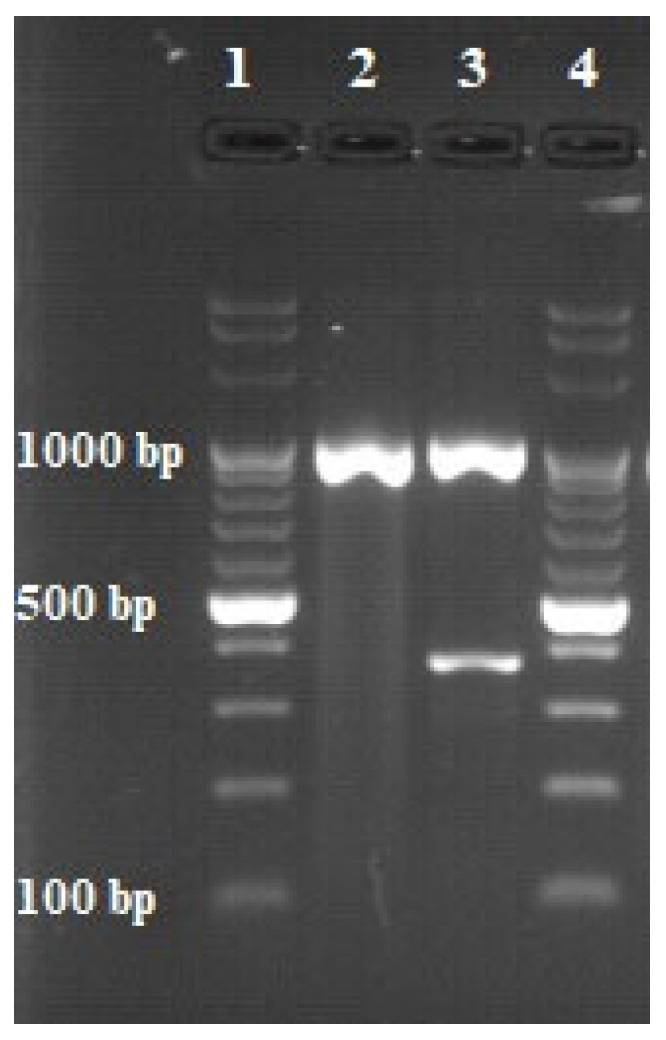
Results of multiplex PCR. 1 and 4—DNA ladder (100 bp); 2—sample; 3—positive control (*M. bovis* DNA).

**Figure 4:**
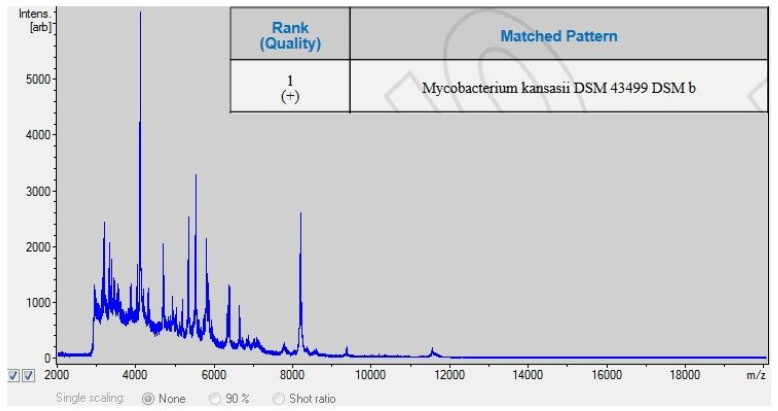
MALDI-TOF results.

The anatomopathological examination of the submandibular lymph nodes showed the physiologically normal structure of the tissue and the absence of lesions indicating infection with bacteria of the genus *Mycobacterium*. After 12 days of incubation, a bacterial isolate (Figure 1) was obtained from culture and determined to be an acid-fast bacilli by staining with the Ziehl–Neelsen method, as indicated by the red color of the stained cells (Figure 2). The use of a multiplex PCR (Figure 3) gave the possibility of determining the bacilli to be nontuberculous, which was indicated by the obtaining of only one DNA band in the position indicating 1030 bp in relation to the DNA ladder and the positive control. Therefore, it prompted the choice of the GenoType Mycobacterium CM test as a method for species identification. Moreover, as a result of exposure to light, the color of the bacterial colonies turned orange, which proved the photochromogenicity of the strain. The GenoType Mycobacterium CM test results indicated that *M. kansasii* was the species isolated from wild boar tissues. At the final stage of the test, the test strip was clearly stained at positions 10 and 12, which, according to the manufacturer’s instructions, is characteristic only for *M. kansasii* [22]. The result was confirmed by MALDI-TOF which gave the same result when used as a confirmatory method. The obtained mass-to-charge ratio of cellular proteins was automatically compared with all those present in the Bruker database and clearly indicated that the obtained protein profile is characteristic for *M. kansasii* (Figure 4). *Mycobacterium kansasii* Hauduroy 1955—NCBI: txid1768 was indicated as NCBI identifier [24]. The test was performed in four replications. However, neither of these methods allow to distinguish between the individual bacteria that are part of the MKC.

## 4. Discussion

MKC poses a grave threat to human health and life; therefore, its presence among free-living animals is of great concern. The results of this study present the first case of isolation of this species from a wild boar in Poland and only the second case in Europe [25] and it is noted to appear equally rarely among other species of free-living animals [12]. The source of the infection of a free-living animal with a pathogen causing disease mainly in humans is a puzzle. Undoubtedly, the omnivorous nature of wild boar and their frequent digging in soil significantly increase the likelihood of transfer the pathogen to the animal organism. Scavenging is a characteristic behavioral trait of wild boar, which additionally increases the risk of infection through alimentation [26,27].

In the analyzed case, the wild boar was shot by a hunter and was apparently healthy. Additionally, during the post-mortem examination, no lesions were found in the internal organs, which retained their physiologically correct structure. A similar case was described by Ghielmetti et al. [28], who isolated *M. kansasii* from cattle that did not show clinical signs of mycobacterial disease and found no macroscopic pathological lesions in the tissues which they tested. The same observations were recorded by Waters et al. [29], who did not find any symptoms of the disease in cattle after experimentally infecting them. Asymptomaticity is a very rare phenomenon, because infection with this pathogen most often causes the animal to become extremely exhausted, which is manifested in respiratory distress, progressive emaciation, lack of appetite, and intestinal stagnation [30,31].

The course of mycobacteriosis caused by *M. kansasii* is most often similar to tuberculosis, which makes diagnosis difficult and, consequently, also impedes the selection of an appropriate treatment regimen [32]. Schafbuch et al. [33] observed an enlargement and mineralization of lymph nodes, as well as numerous nodules in the lungs, liver, spleen, and kidneys in a Vietnamese pot-bellied pig (*Sus scrofa domesticus*), which is closely related to the wild boar. The contrasting picture in the described case of wild boar mycobacteriosis is a puzzle, but it may result from possible elimination of the microorganism by the immune system of the host. Among the pathological lesions described in the literature, there is also an excess of clean peritoneal fluid, a significant enlargement and irregularity of form of the lymph nodes, and numerous irregular, pale changes in the liver and lungs [34,35,36]. A cutaneous form of the disease may occasionally occur, as exemplified by the case of a cat infected with *M. kansasii*. A 1-year-old female domestic shorthair cat presented with anorexia, depression, and weight loss, accompanied by multifocal nodules affecting the face and occasional ulceration affecting the ears, periocular areas, and nasal planum [37]. Infection with obvious clinical signs and coinciding lymphadenitis or lung lesions have been reported in rhesus monkeys (*Macaca mulatta*), cattle (*Bos taurus*), llamas (*Lama glama*), alpacas (*Vicugna pacos*), goats (*Capra hircus*), camels (*Camelus*), and domestic pigs (*Sus scrofa f. domestica*) [33,38]. The extensiveness of the range of animal species susceptible to infection with MKC poses a high risk of uncontrolled transmission of the pathogen in the environment, as is often the case with *M. bovis* [39]. In addition, because cattle and wild boars frequently share feeding areas, the pathogen may be transmitted from boars to cattle, which in turn may lead to the uncontrolled spread of the disease. Unfortunately, there is no effective prophylaxis to protect livestock in the case of the spread of this microorganism [40]. Moreover, it has been proven that this pathogen can be found in the milk of infected individuals [41]. Mycobacteriosis caused by MKC may also misrepresent the individual’s disease status in skin tests and other assays used in the diagnosis of tuberculosis in animals and humans and produce false positive results. This fact significantly complicates the treatment process in humans and is the reason for wrong decisions to slaughter the animal [42,43,44].

The presented case of MKC member isolation from the tissues of a wild boar can be contrasted with the results of research by Ronai et al. [45], who by examining 49 strains of mycobacteria isolated from this animal species, ruled out the presence of *M. kansasii* in the studied population. Similar research results were achieved by Garcia-Jimenez et al. [25] investigating submandibular lymph node samples from 1249 wild boars, because they isolated 219 nontuberculous mycobacteria strains but only identified the strain as *M. kansasii* in one case. The results of these studies prove that microorganisms belonging to MKC is a mycobacterium that is very rarely responsible for wild boar mycobacteriosis, which makes the analyzed case extremely interesting. Taking into account the popularity of hunting wild boar, potential poaching and the fact that wild boar is living more and more close to humans, there is a risk of transmission of the pathogen to humans and in consequence to farm and domestic animals [46]. An examination of the genetic relationship of *M. bovis* strains, which have a similar range of hosts to MKC, was carried out by Casalinuovo et al. [47] and they proved that the strain obtained from wild boar had also been isolated from cattle and a human within the 6 years prior to the boar sample’s investigation. The infected human was a poacher, and his infection was likely to have occurred through the ingestion of meat subjected to improper thermal treatment or by contact of an injured hand with tissue during the evisceration of the animal. Knowledge of the MKC presence among the wild boar population can help to make the public aware of the dangers of dressing wild animals or consuming their tissue in ignorance of safe procedures. To control the prevalence of MKC in the wild boar population, it is necessary to conduct regular examinations of individuals shot by hunters. It should also be borne in mind that the infection of wild boar with this species of NTM will not always be synonymous with the occurrence of internal organ lesions, which should additionally induce hunters to take appropriate precautions. Tuberculosis-like lesions of the lymph nodes, lungs, and liver may be the result of diseases caused by many other bacterial species, including *Rhodococcus equi*, *Corynebacterium silvaticum*, *Streptococcus dysgalactiae*, or *Staphylococcus aureus*; therefore, appropriate differential diagnosis should be performed. *R. equi* infects many species of farm and wild animals and can also cause purulent pneumonia and granulomatous lesions. Moreover, many scientists believe that animals infected with *Mycobacterium* spp. are more likely to be coinfected with *R. equi* [48,49,50,51]. In the case of isolation of a bacterial strain from samples of veterinary origin, quick species identification of the pathogen is essential. Undoubtedly, the use of MALDI-TOF MS makes it possible. Literature sources show that MALDI-TOF MS has a high concordance rate to the reference methods and because of its rapidness, cost-effectiveness, and high throughput, it represents a valid diagnostic tool for identification of NTM species in veterinary medicine [7].

## 5. Conclusions

The aim of the study was to exclude or confirm the infection of wild boar living in Poland by bacteria of the genus *Mycobacterium*. The test results clearly showed that the animal was infected with a pathogen belonging to MKC. These pathogens are mainly responsible for human diseases, and cases of mycobacteriosis in free-living animals are extremely rare. The described case of a wild boar infection by MKC is the first documented case in Poland and the second in Europe and brings to light the presence of this pathogen, which is dangerous to life for animals and humans, among free-living animals. This finding is of great concern. The presented results may contribute to making the affected section of the public aware of the dangers of dressing wild animals or consuming their meat in ignorance of safe procedures.

## Figures and Tables

**Table 1:** Starter sequences used in the reaction.

Identification	Target DNA	Primer	Sequence (5′–3′) *	Sequence Length (bp)
*Mycobacterium* genus	16 S rRNA	Mycgen-F	AGA GTT TGA TCC TGG CTC AG	1030
		Mycgen-R	TGC ACA CAG GCC ACA AGG GA	
*Mycobacterium tuberculosis* complex	MPB70	TB1-F	GAA CAA TCC GGA GTT GAC AA	372
		TB1-R	AGC ACG CTG TCA ATC ATG TA	

* The primer sequences were obtained from the European Reference Laboratory for Bovine Tuberculosis in Madrid, Spain.

**Table 2:** Reaction components.

Reagent	Concentration	Volume (µL)
Nuclease-free water	-	3.4
Multiplex PCR Master Mix	2×	12.5
Mycgen-F and -R primers	35 ng/µL	1.5
TB1-F and -R primers	20 ng/µL	2.5
Mg^2+^	25 mM	0.1
DNA	-	5
Total volume	25	

**Table 3:** PCR conditions.

Cycle	Time	Temperature
Initial denaturation	10 min	95 °C
Denaturation	30 s	95 °C
Annealing	2 min	65 °C
Elongation	3 min	72 °C
Final elongation	10 min	72 °C

## Data Availability

No new data were created or analyzed in this study. Data sharing is not applicable to this article.
